# Inhibition of OXPHOS induces metabolic rewiring and reduces hypoxia in murine tumor models

**DOI:** 10.1016/j.ctro.2024.100875

**Published:** 2024-10-14

**Authors:** Daan F. Boreel, Anne P.M. Beerkens, Sandra Heskamp, Milou Boswinkel, Johannes P.W. Peters, Gosse J. Adema, Paul N. Span, Johan Bussink

**Affiliations:** a Radiotherapy and OncoImmunology Laboratory, Department of Radiation Oncology, Radboudumc, Nijmegen, the Netherlands; b Department of Medical Imaging, Radboudumc, Nijmegen, the Netherlands

**Keywords:** Hypoxia, Metabolism, OXPHOS, IACS-010759, Metformin, Atovaquone

## Abstract

**Introduction:**

Tumor hypoxia is a feature of many solid malignancies and is known to cause radio resistance. In recent years it has become clear that hypoxic tumor regions also foster an immunosuppressive phenotype and are involved in immunotherapy resistance. It has been proposed that reducing the tumors’ oxygen consumption will result in an increased oxygen concentration in the tissue and improve radio- and immunotherapy efficacy. The aim of this study is to investigate the metabolic rewiring of cancer cells by pharmacological attenuation of oxidative phosphorylation (OXPHOS) and subsequently reduce tumor hypoxia.

**Material and methods:**

The metabolic effects of three OXPHOS inhibitors IACS-010759, atovaquone and metformin were explored by measuring oxygen consumption rate, extra cellular acidification rate, and [^18^F]FDG uptake in 2D and 3D cell culture. Tumor cell growth in 2D cell culture and hypoxia in 3D cell culture were analyzed by live cell imaging. Tumor hypoxia and [^18^F]FDG uptake *in vivo* following treatment with IACS-010759 was determined by immunohistochemistry and *ex vivo* biodistribution respectively.

**Results:**

*In vitro* experiments show that tumor cell metabolism is heterogeneous between different models. Upon OXPHOS inhibition, metabolism shifts from oxygen consumption through OXPHOS towards glycolysis, indicated by increased acidification and glucose uptake. Inhibition of OXPHOS by IACS-010759 treatment reduced diffusion limited tumor hypoxia in both 3D cell culture and *in vivo*. Although immune cell presence was lower in hypoxic areas compared with normoxic areas, it is not altered following short term OXPHOS inhibition.

**Discussion:**

These results show that inhibition of OXPHOS causes a metabolic shift from OXPHOS towards increased glycolysis in 2D and 3D cell culture. Moreover, inhibition of OXPHOS reduces diffusion limited hypoxia in 3D cell culture and murine tumor models. Reduced hypoxia by OXPHOS inhibition might enhance therapy efficacy in future studies. However, caution is warranted as systemic metabolic rewiring can cause adverse effects.

## Highlights

• OXPHOS inhibition induces metabolic rewiring in tumor cells, spheroids and normal tissues.
• OXPHOS inhibition reduces tumor hypoxia in tumor spheroids and *in vivo*.
• Caution is warranted as systemic metabolic rewiring can cause adverse effects.
• OXPHOS is a potential therapeutic target to alleviate hypoxia and increase radio- and immunotherapy efficacy.

## Introduction

Hypoxia is a common feature of many solid malignancies and a prognostic biomarker that impacts outcome of cancer patients [1]. Moreover, tumor hypoxia is a well described cause for radiotherapy resistance [2]. Additionally, the tumor immune microenvironment can be greatly influenced by tumor hypoxia [3]. For instance, hypoxia is associated with T-cell exhaustion and an increased presence of tumor associated macrophages, leading to increased immune escape by cancer cells [4], [5]. These features of the hypoxic tumor microenvironment seem to contribute to immunotherapy resistance, as observed in both mouse tumor models and cancer patients [6], [7], [8]. Therefore, increased resistance of hypoxic tumors to radiotherapy and immunotherapy makes tumor hypoxia a major obstacle in the efficacy of cancer therapy.

Hypoxia can arise due to temporal occlusion of tumor vasculature known as acute hypoxia [9]. However, a substantial part of hypoxia arises when the oxygen demand of tissue, through oxidative phosphorylation (OXPHOS), exceeds the supply leading to a more chronic hypoxia [10]. Chronic or diffusion-limited hypoxia can be observed at an increased distance to the perfused tumor vasculature and has many biological and metabolic consequences, mainly through HIF-1α stabilization, but also by other pathways like the unfolded protein response [11], [12]. One of these metabolic consequences of hypoxia is an upregulation of glycolysis that will lead to elevated lactate and pyruvate levels [13]. Another metabolic pathway that is upregulated, through HIF-1α stabilization, is the pentose phosphate pathway, making use of glycolytic intermediate glucose-6-phosphate [14]. These changes in metabolism caused by hypoxia are examples of how tumor cells can adapt to their environment by inducing a metabolic rewiring from OXPHOS to glycolysis or the other way around [15].

The majority of strategies countering hypoxia-mediated treatment resistance, such as oxygen breathing and administration of vasodilating drugs, lead to a short lasting, temporal decrease in tissue hypoxia, likely too short to result in a sustained effect on the local immune cell repertoire [16]. To achieve a more durable response on hypoxia it is proposed to reduce the oxygen consumption in tumor tissue and thereby increase tissue oxygenation [10]. This approach has already shown to decrease hypoxia and increase radiotherapy or immunotherapy efficacy in several mouse models using various OXPHOS inhibitors [17], [18], [19]. In this study we explored the consequences of metabolic reprogramming by OXPHOS inhibition in *in vitro* 2D cell culture, 3D spheroids and *in vivo* with syngeneic immunocompetent mouse tumor models using the mitochondrial complex I inhibitors IACS-010759 and metformin, and the mitochondrial complex III inhibitor atovaquone.

## Material and methods

### *In vitro* assays

#### 2D and 3D cell culture

MOC1 (provided by Dr. R. Uppaluri, Dana-Farber Cancer Institute, Boston) and MOC1.3D5 murine oral cancer cells were cultured in 2:3 IMDM, 1:3 Ham’s F-12 nutrient mix with 5 % Fetal Calf Serum (FCS), 1 % penicillin–streptomycin (all Gibco), 5 ng/mL Epidermal Growth Factor (EMD Millipore), 40 ng/mL hydrocortisone and 5 μg/mL insulin (both Sigma Aldrich) [20]. MC38 (Kerafast) murine colon carcinoma cells were cultured in DMEM Glutamax with 10 % FCS, 1 % sodium pyruvate, 1 % nonessential amino acids (NEAA), and 1 % penicillin–streptomycin (all Gibco). B16ova murine melanoma cells (provided by Dr. K.L. Rock, Dana-Farber Cancer Institute, Boston) were cultured in MEM with 5 % FCS, 2 % sodium bicarbonate, 1.5 % MEM vitamins, 1 % sodium pyruvate, 1 % NEAA, 1 % antibiotic/antimycotic, 0.1 % β-mercaptoethanol, 1 mg/mL G418, and 60 μg/mL hygromycin (all Gibco). GL261 murine glioblastoma cells (provided by Dr. U. Herrlinger, University of Bonn, Bonn) were cultured in IMDM, 10 % FCS, 0.1 % β-mercaptoethanol, and 1 % penicillin–streptomycin. When cultured under low glucose conditions all cells were cultured in DMEM with 10 % FCS, 1 % sodium pyruvate, 1 % NEAA, 1 % penicillin–streptomycin and 1.5 mM glucose. MC38, B16ova and MOC1.3D5 cells containing a HIF1α responsive element (HRE)-eGFP-ODD construct were generated as described before [21], [22]. To form spheroids, 10,000 cells per well were seeded in U-bottom ultra-low attachment plates (Corning) in 2.5 % Matrigel (Corning) and centrifuged for 10 min at 1000 rpm [23]. Cells and spheroids were cultured at 37 °C and 5.0 % CO_2_. All cell lines used were regularly tested for mycoplasma and are mycoplasma-free.

#### Flow cytometry analysis

For flow cytometry analysis, cells were incubated with viability dye eFluor^TM^ 780 (eBioscience) in PBS for 15 min at 4 °C to distinguish living and dead cells. Intracellular staining was performed using the Cytofix/Cytoperm^TM^ Fixation/Permeabilization Kit (BD) according to the manufacturer’s protocol and using the following primary antibodies, secondary antibodies and isotype controls: rabbit phospho-AMPK alpha-1,2 (Thr183, Thr172), mouse AMPK alpha-1, goat anti-mouse Alexa Fluor 488 and donkey anti-rabbit Alexa Fluor 488 (all Invitrogen) and mouse IgG1, k and rabbit IgG (both Biolegend). Cells were washed in PBA (0.05 % sodium azide in PBS) and measured by flow cytometry (FACSCanto II, BD). Data were analyzed using FlowJo V10.7 (Tree Star).

#### Seahorse metabolic assay

Metabolic activity of cells was determined by measuring oxygen consumption and lactate production using the Seahorse XF-96 Extracellular Flux Analyzer (Agilent). 40,000 cells/well were seeded in Seahorse XF-96 microplates (Agilent) and were incubated overnight with OXPHOS inhibitors IACS-010759 (Selleckchem), atovaquone or metformin, or DMSO control (all Sigma). Before measurement the medium was replaced with Seahorse medium (8.3 g DMEM powder, 0.016 g phenol (both Sigma) and 1.85 g NaCl (Merk) in sterile water supplemented with 11 mM D-glucose (mito stress test), 2 mM L-glutamine and 1 mM pyruvate (all Sigma), pH 7.4) and incubated for 1 h at 37 °C and 0 % CO_2_. Mito Stress Test was performed using oligomycin (1 µM), FCCP (1 µM) and antimycin (2.5 µM) with rotenone (1.25 µM) (all Sigma) basal respiration was calculated according to manufacturer’s protocol. Glyco Stress Test was performed using D-glucose (11 mM), oligomycin (1 µM) and 2-deoxy-D-glucose (22 mM) (all Sigma) glycolysis was calculated according to manufacturer’s protocol. Data were analyzed using Wave 2.3.0 (Agilent).

#### Live cell and spheroid imaging

*In vitro* cell growth and fluorescence was measured using the IncuCyte ZOOM Live-Cell Analysis System (Essen BioScience). Cells were seeded in 96-wells plates (Corning) and fluorescence and brightfield images of cells or spheroids were retrieved and confluency was calculated as described before [14]. After 72 h metabolic activity of samples was measured with the Cell Counting Kit-8 (CCK-8) to investigate cytotoxicity (Sigma-Aldrich) according to manufacturer’s protocol. Live cell imaging at hypoxic conditions was performed at 1 % O_2_ in a Whitley H35 Workstation (Don Whitley Scientific).

#### [^18^F]FDG uptake assay

[^18^F]fluorodeoxyglucose (FDG) (150 kBq/well) (Radboud Translational Medicine) was added to medium of cells cultured to confluency in 6-well plates (Corning) or spheroids cultured in 96-well plates, followed by incubation for 2 h at 37 °C and 5.0 % CO_2_. After incubation cells and spheroids were washed with PBS and cell-associated activity was measured using a γ-counter (2480 Wizard, Perkin-Elmer). Percentage uptake was determined relative to the added activity.

### Animal experiments

Female C57BL/6 mice (10–12 weeks, Charles River) were inoculated subcutaneously with 0.5 × 10^6^ B16ova or MC38 in phosphate-buffered saline (PBS) or with 0.5 × 10^6^ MOC1.3D5 cells in 1:3 matrigel/PBS (BD Bioscience) on the right hindleg. Mice were housed in individually ventilated cages with a filter top (Green line IVC, Tecniplast), with food and water present *ad libitum*. Experiments started when tumors reached an average size of approximately 100 mm^3^ and mice were randomized based on tumor size. To mark hypoxia and perfusion, all mice were injected with pimonidazole (J. A. Raleigh, Department of Radiation Oncology, University of North Carolina) intraperitoneally 1 h prior to sacrifice (80 mg/kg), and Hoechst 33,342 (Sigma) at 1 min prior to sacrifice, i.v. (15 mg/kg), respectively.

#### Reducing tumor hypoxia by OXPHOS inhibition *in vivo*

To investigate if OXPHOS inhibition can reduce tumor hypoxia *in vivo,* mice bearing MC38 or MOC1.3D5 tumors received oral IACS-010759 (10 mg/kg) or vehicle control (0.5 % methylcellulose) treatment for 4 consecutive days (n = 6 per group). 5 h post the last IACS-010759 treatment, mice were sacrificed and the tumor was dissected and snap frozen for immunohistochemical (IHC) analysis of hypoxia and immune cell infiltration. Of mice bearing MOC1.3D5 tumors, blood was collected directly after sacrifice for lactate measurement.

#### Lactate measurement

Mouse blood was left to clot for 1 h at 4 °C and centrifuged at 2000 x G for 10 min to collect serum. Samples were deproteinized with 13.7 % perchloric acid and neutralized with 4 N NaOH. Lactate concentrations were determined in deproteinized serum through the conversion of lactate by lactate oxidase (Merck). The subsequent oxidation of Amplex Red reagent (ThermoFisher Scientific) to resorufin via HRP (ThermoFisher Scientific) was measured as a fluorescent signal with an HTX microplate reader (Biotek).

#### [^18^F]FDG uptake following OXPHOS inhibition *in vivo*

To investigate glucose uptake in tumor and normal tissues following OXPHOS inhibition, mice bearing B16ova tumors received oral IACS-010759 or vehicle control using the same treatment schedule as in previous studies (n = 6 per group). Blood glucose was determined using an Accu Check Aviva (Roche). Mice were anesthetized 4 h post the last IACS-010759 treatment using isoflurane and subsequently intravenously injected with [^18^F]FDG (0.2 mL, 10.0 ± 0.3 MBq). Mice were maintained under anesthesia at 37 °C until sacrifice to reduce excessive muscle uptake (total duration of anesthesia was 80 min). Mice were particularly sensitive to isoflurane when treated with IACS-010759, indicated by a low respiration rate (approx. 10–20 per minute). This is in line with previous observations and necessitated close monitoring of the animal welfare during anesthesia [24]. Because of the poor general condition of these mice and their low respiration rate, we cannot draw any conclusions on the effect of OXPHOS inhibition on [^18^F]FDG uptake from this study. Therefore, in the subsequent study in B16ova tumor-bearing mice, no anesthesia was applied. Following intravenous injection of [^18^F]FDG (0.2 mL, 9.9 ± 0.3 MBq), mice were kept awake without access to food until sacrifice. After sacrifice (80 min post injection), tumor (snap frozen), blood, brain, heart, lung, liver, kidney, lymph nodes, spleen, muscle, stomach, small intestine and colon were dissected, weighed and activity was measured using a γ-counter.

#### Immunohistochemistry

For IHC analyses, frozen tumors were sectioned (5 μm) and mounted on poly-l-lysine coated slides and fixed in ice-cold acetone for 10 min. IHC staining was performed to evaluate leukocytes (CD45.2), hypoxia (pimonidazole), perfusion (H33342), and vessels (9F1), as previously described [3], [25]. Whole-tissue section grayscale images (pixel size, 2.59 × 2.59 μm) for vessels, perfusion, pimonidazole and CD45.2 were obtained and subsequently converted into binary images as previously described [25]. Thresholds for segmentation of the fluorescent signals were manually set above the background signal for each individual marker. Binary images were used to calculate the CD45.2, pimonidazole or perfused fraction relative to the total tumor area. The quantification of pimonidazole and CD45.2 fractions at different distances to perfused vasculature was done as previously described [26].

### Statistical analysis

Statistical analyses were performed using GraphPad Prism (version 8.0). Unpaired *t*-test was used to compare groups. When samples were not normally distributed a Mann-Whitney test was used. One-way ANOVA was used to compare > 2 groups. *P* values of 0.05 or less were considered significant. Results are expressed as mean ± SD.

## Results

### Syngeneic mouse tumor models are metabolically heterogenous and inhibition of OXPHOS reduces oxygen consumption

**Fig. 1:**
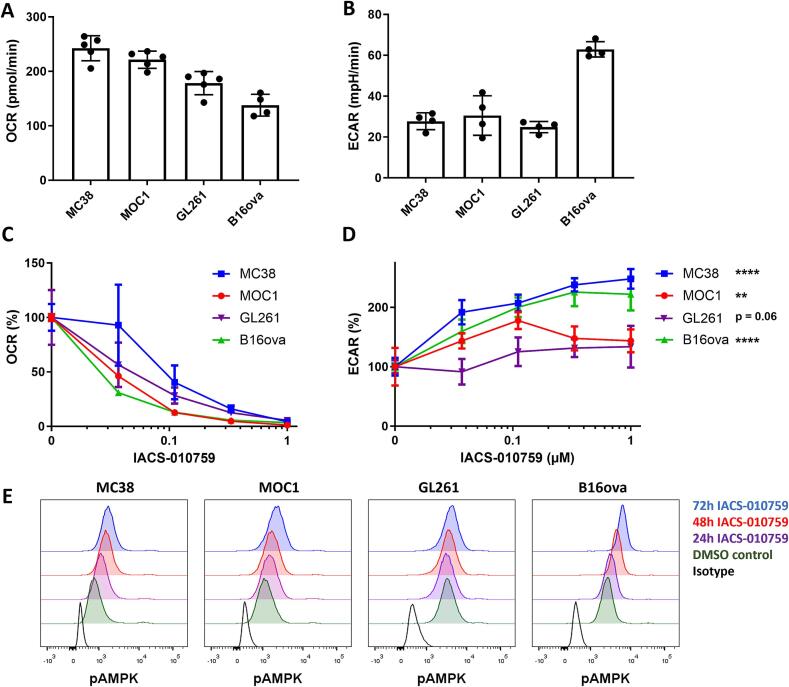
Murine tumor cells use OXPHOS, which can be inhibited by IACS-010759. (A) Basal respiration (OCR) of several murine cancer cell lines. (B) Glycolysis (ECAR) of several murine cancer cell lines. (C) Basal respiration (% OCR) of several murine cancer cell lines treated with IACS-010759 for 24 h. (D) Glycolysis (% ECAR) of several murine cancer cell lines treated with IACS-010759 for 24 h. (E) Phospo-AMPK abundance in several murine cancer cell lines treated untreated or treated with IACS-010759 (1.0 μM) for 24, 48 and 72 h.

We characterized the metabolism of several murine tumor cell lines by measuring the oxygen consumption rate (OCR) and extracellular acidification rate (ECAR). Basal OCR of these cells differed substantially, with MC38 and MOC1 showing the highest basal OCR, while GL261 and B16ova showed lower oxygen consumption (Fig. 1A). Interestingly, B16ova had a substantially higher glycolytic rate compared to the other cell lines, while the other cells showed an equal level of glycolysis (Fig. 1B). Subsequently, we showed that the potent mitochondrial complex I inhibitor IACS-010759 reduced OCR of all cell lines in a dose dependent manner (Fig. 1C). This effect was also observed, but at much higher doses, when using the mitochondrial complex III inhibitor atovaquone or mitochondrial complex I inhibitor metformin (Supplementary fig. S1). When cell lines were treated with the most potent inhibitor IACS-010759, that was used in subsequent experiments, glycolysis was increased in various levels in all cell lines except for GL261 and most notably in B16ova (Fig. 1D).

One of the possible mechanisms by which metabolic rewiring is mediated in tumor cells is by the phosphorylation of 5′ adenosine monophosphate-activated protein kinase (AMPK), an intracellular sensor of energetic stress [27]. Treatment of cells with IACS-010759 up to 72 h resulted in a time dependent increase in the presence of phosphorylated AMPK (pAMPK) measured by flow cytometry. This was most pronounced in B16ova and absent in GL261. The presence of un-phosphorylated AMPK was not, or only marginally increased, suggesting inhibition of OXPHOS can lead to an increased phosphorylation of AMPK and a subsequent increase of glycolysis (Supplementary fig. S2).

### Cancer cell vulnerability to OXPHOS inhibition is both glucose- and oxygen-dependent

**Fig. 2:**
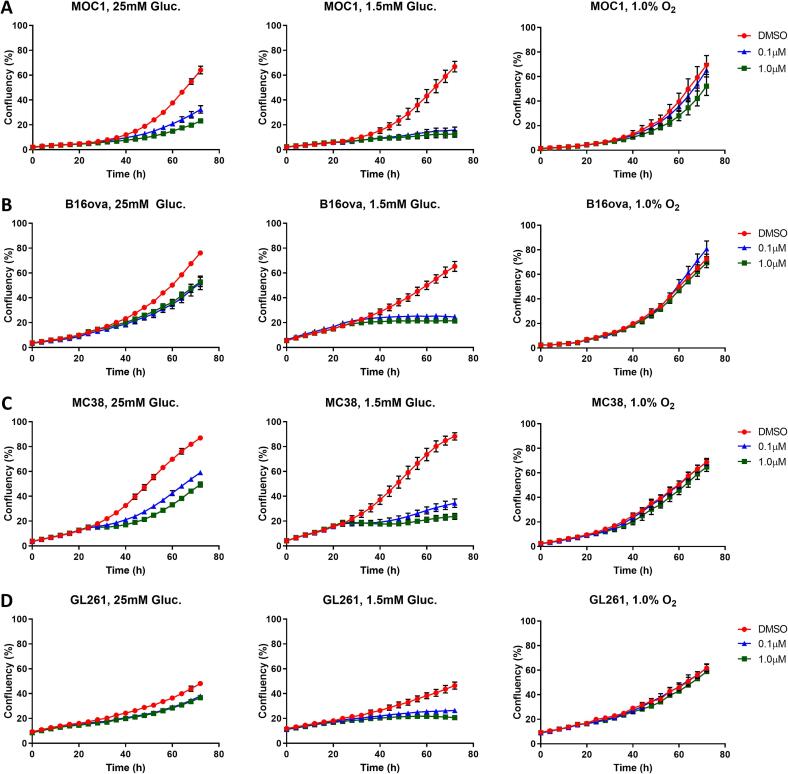
Decrease of cell growth by OXPHOS inhibition is nutrient and oxygen dependent. Cell growth of (A) MOC1, (B) B16ova, (C) MC38 and (D) GL261 under high glucose (25 mM), low glucose (1.5 mM) and hypoxic (1.0 % O_2_, 25 mM Glucose) conditions. IACS-010759 or DMSO were added to culture medium at 24 h.

In the majority of studies, *in vitro* cell culture-based experiments are performed using high glucose medium (25 mM glucose), while physiological glucose conditions are often much lower (0.5–5 mM, [28]). To test if physiological glucose and oxygen concentrations alter vulnerability of cells to OXPHOS inhibition, we monitored cell growth at different glucose and oxygen concentrations. Under high glucose concentrations, all cell lines exhibited an inhibition of cell growth following IACS-010759 treatment (Fig. 2). When cultured under more physiological (1.5 mM) glucose conditions, increased inhibition of cell growth was observed for the 4 cell lines after OXPHOS inhibition. Using a CCK-8 assay, metabolic activity at 48 h post IACS-010759 treatment was measured on the same cells (Supplementary fig. S3). Cytotoxicity significantly increased in all tested cell lines under 25 mM glucose conditions. This effect was even more pronounced under physiological (1.5 mM) glucose. In contrast, under hypoxic conditions (1.0 % oxygen) an inhibitory effect on cell growth and proliferation by IACS-010759 was almost completely absent in all cells tested. This data indicates that cancer cell metabolism is plastic and cells change dependency on OXPHOS or glycolysis in response to both glucose and oxygen availability.

### Inhibition of OXPHOS reduces hypoxia and promotes glucose uptake in tumor spheroids

**Fig. 3:**
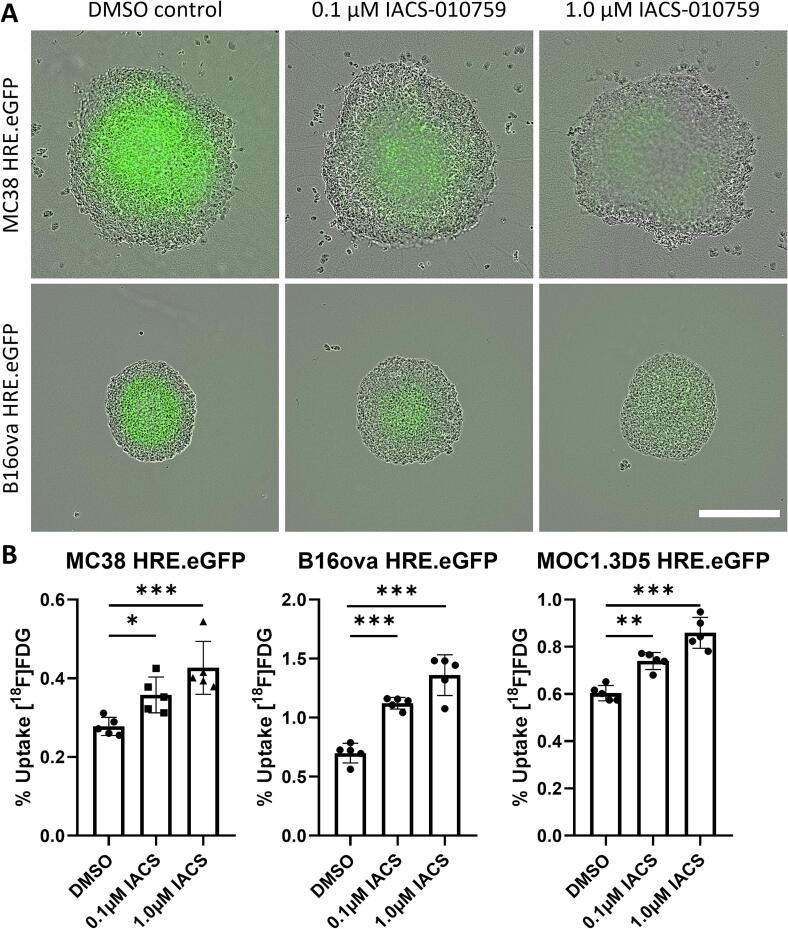
OXPHOS inhibition reduces hypoxia and promotes glucose uptake in tumor spheroids. (A) Fluorescent images of MC38 HRE.eGFP and B16ova HRE.eGFP spheroids 3 h post treatment with IACS-010759 or DMSO. Scalebar represents 500 μm. (B) Uptake of [^18^F]FDG in MC38 HRE.eGFP, B16ova HRE.eGFP and MOC1.3D5 HRE.eGFP spheroids 3 h post treatment with IACS-010758 or DMSO.

Diffusion limited hypoxia is a result of oxygen consumption in tumor tissue and is not present in 2D cell culture, but is present in tumor spheroids exceeding a diameter of approximately 450 µm [29]. To visualize hypoxia *in vitro*, tumor cell spheroids were formed using MC38, B16ova and MOC1.3D5 cells containing an HRE-eGFP-ODD reporter construct. Hypoxia in MOC1.3D5 spheroids containing an HRE-eGFP ODD construct could not be visualized due to the weak signal. MC38 and B16ova spheroids, however, showed a clear eGFP signal in hypoxic cores 24 h after formation (Fig. 3A). Three h following treatment with IACS-010759, the eGFP signal disappeared in a dose dependent manner, showing alleviation of hypoxia (Fig. 3A). A similar effect, but at higher doses and at a later timepoint (24 h vs. 3 h) was observed using the more moderately effective OXPHOS inhibitors atovaquone and metformin (Supplementary fig. S4).

When cells were treated with IACS-010759 in 2D cell culture for 3 h, [^18^F]FDG uptake was increased independent of dose (Supplementary fig. S5). [^18^F]FDG uptake by tumor spheroids, that showed decreased hypoxia, was increased in a dose dependent manner following 3 h of IACS-010759 treatment (Fig. 3B). These results suggest that a shift towards increased glucose uptake is necessary to ensure sufficient levels of ATP by means of glycolysis, not only in 2D cell culture, but also in more complex 3D cell culture.

### Inhibition of OXPHOS reduces tumor hypoxia *in vivo*

**Fig. 4:**
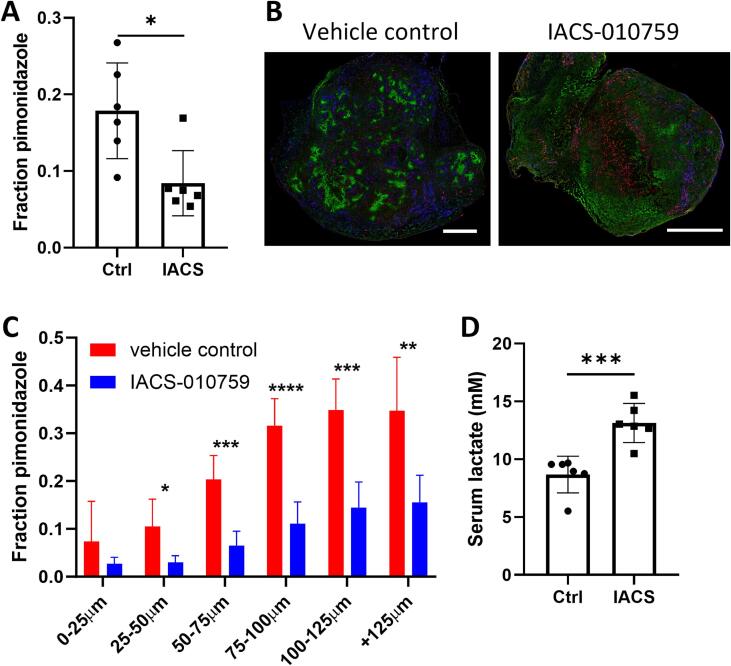
Inhibition of OXPHOS reduces tumor hypoxia and increases serum lactate in MOC1.3D5 tumors. (A) Fraction pimonidazole positive area on MOC1.3D5 whole tumor sections of mice treated with IACS-010759 (10 mg/kg) or vehicle control for 4 consecutive days, n = 6. (B) Color merges of A showing hypoxia (pimonidazole, *green*), vasculature (9F1, *red*) and perfused area (Hoechst 33342, *blue*), 10x magnification, scalebar represents 1 mm. (C) Pimonidazole fractions at binned distances to perfused vasculature (D) Serum lactate levels (mM).

To test if inhibition of OXPHOS can reduce hypoxia *in vivo*, mice bearing MOC1.3D5, MC38 or B16ova tumors were treated with the most potent and specific inhibitor IACS-010759 (10 mg/kg) for 4 consecutive days before sacrifice [30]. Pimonidazole staining on MOC1.3D5 sections revealed a significant reduction in hypoxic fraction compared to mice treated with the vehicle control (Fig. 4A-B). To specifically assess the effect of OXPHOS inhibition on diffusion limited hypoxia, the pimonidazole fraction at binned distances to perfused vasculature was evaluated. In line with the hypothesis of alleviating hypoxia by attenuating oxygen consumption, hypoxia was reduced at distances to the perfused tumor vasculature (Fig. 4C). Moreover, serum lactate was significantly increased in mice treated with IACS-010759 compared to vehicle control treated mice, indicating a shift to glycolytic metabolism (Fig. 4D). A similar trend in reduced hypoxia was observed in mice bearing MC38 tumors, although limited overall tumor hypoxia in untreated tumors made the effect less apparent (Supplementary Fig. S6A). B16ova tumors did not show a decreased hypoxic fraction on whole tumor sections (Supplementary Fig. S6C). When examining hypoxia relative to perfused vasculature, MC38 and B16ova sections revealed decreased hypoxia at binned distances to the vasculature, indicating a decrease in diffusion limited hypoxia induced by OXPHOS inhibition (Supplementary Fig. S6B and D).

### Inhibition of OXPHOS induces systemic metabolic changes *in vivo*

**Fig. 5:**
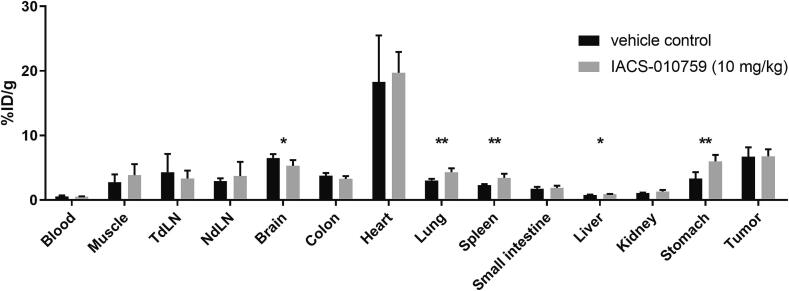
Ex vivo biodistribution of [^18^F]FDG in mice bearing B16ova tumors treated with IACS-010759 (10 mg/kg) or vehicle control for 4 consecutive days. n = 5. Tumor draining lymph node; TdLN, Non-draining lymph node; NdLN.

The effect of OXPHOS inhibition on glucose metabolism of tumors and normal tissues *in vivo* was evaluated in B16ova-tumor bearing mice using [^18^F]FDG uptake studies. Biodistribution results showed an increased [^18^F]FDG uptake in mice treated with IACS-010759 in stomach (p = 0.003), lung (p = 0.003), spleen (p = 0.005) and liver (p = 0.04) compared to mice treated with the vehicle control (Fig. 5 and Supplementary Table S1). [^18^F]FDG uptake in the brain was reduced (p = 0.04) in these mice. B16ova tumor, the model that showed the greatest increase in glucose uptake *in vitro*, and other selected organs did not show a significant difference in [^18^F]FDG uptake. No differences in blood glucose were observed in mice treated with IACS-010759 or vehicle control as measured before [^18^F]FDG administration (Supplementary Table S1).

### Immune cell presence is not altered following short-term OXPHOS inhibition

**Fig. 6:**
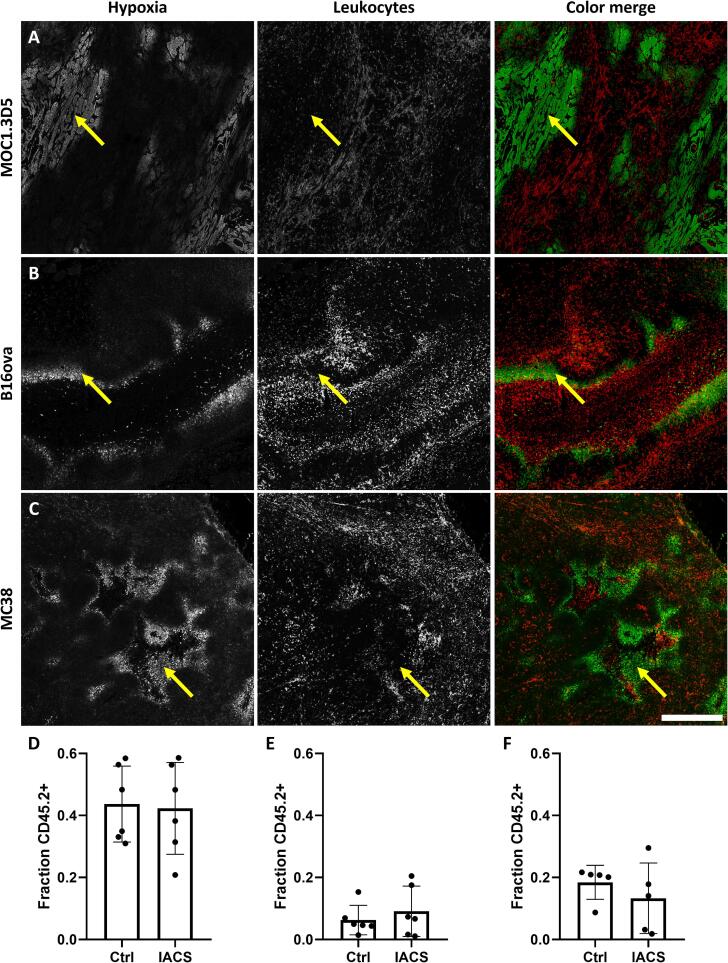
Immune cell presence is not altered following 4 days of OXPHOS inhibition. Gray value images and a color merge of (A) MOC1.3D3, (B) B16ova and (C) MC38 tumor tissue showing hypoxia (pimonidazole, green) and leukocytes (CD45.2, red). Yellow arrows indicate hypoxic areas with less infiltration of immune cells, 10x magnification, scalebar represents 500 μm. Fraction CD45.2 positive cells is not altered when treated with IACS-010759 (10 mg/kg) on (D) MOC1.3D5 (n = 6), (E) B16ova (n = 6) or (F) MC38 (n = 5) tumor sections for 4 consecutive days.

To confirm that hypoxic areas are less infiltrated with immune cells in the murine tumor models used in this study, leukocytes and hypoxia were visualized on tumor sections by IHC. A decreased infiltration of CD45.2^+^ leukocytes was observed in hypoxic areas stained by pimonidazole compared to the directly adjacent viable tumor tissue (Fig. 6A-C). This effect was most prominently present in MOC1.3D5 and B16ova tumors, but also observed in MC38 tumor tissue. These observations underline the potential for increasing the anti-tumor immune response by alleviation of hypoxia in these tumor models. However, while a reduction in hypoxia was observed, immunohistochemical staining for CD45.2 showed no difference in the presence of CD45.2^+^ leukocytes when treated with IACS-010759 for 4 days in all tumor models tested on whole tumor sections (Fig. 6D-F). In addition, the localization of immune cells relative to perfused vessels was not altered by the treatment schedule used in this study (Supplementary fig. S7), suggesting longer hypoxia-alleviation may be necessary.

## Discussion

Tumor hypoxia, commonly present in solid malignancies, plays an important role in the resistance to radio- and immunotherapies [6], [7], [31]. Diffusion limited hypoxia is driven by oxygen consumption, suggesting that oxygen consumption is present in tumors as opposed to the longstanding notion that tumors are primarily glycolytic, even when oxygen is present (i.e. aerobic glycolysis, Warburg effect) [32]. This is in accordance with the high level of oxygen consumption we have measured in some tumor cell lines and the level of hypoxia in B16ova and MC38 spheroids. To overcome therapy resistance, the pharmacological inhibition of OXPHOS has been suggested. This would reduce the OCR and thereby decrease tumor hypoxia, potentially resulting in increased radiosensitivity and a more immune permissive microenvironment [17], [33]. In this study we show that of the tested mitochondrial complex inhibitors, IACS-010759 most potently decreases oxygen consumption, pushes metabolism towards glycolysis, and consequentially decreases hypoxia both *in vitro* and *in vivo*.

Inhibition of OXPHOS by IACS-010759 resulted in a decrease of hypoxia in both tumor spheroids as well as in several murine tumor models in this study. This significant reduction of hypoxia in tumor spheroids was quantified using the same cell lines by Beerkens et al. [22]. While on MOC1.3D5 and MC38 whole tumor sections a decrease in hypoxia (pimonidazole fraction) is observed, such an overall reduction is not present in B16ova tumors. This could be caused by heterogeneity in tumor tissue in B16ova compared to the other models, resulting in more hypoxia and necrosis as the balance between diffusion limited hypoxia relative to perfusion limited hypoxia is different. Closer analysis of hypoxia at binned distances to perfused tumor vasculature shows that diffusion limited hypoxia in B16ova, like in other models, is reduced following IACS-010759 treatment. When investigating hypoxia in different tumor subregions using hypoxia PET/CT following OXPHOS inhibition, spatial differences in hypoxia reduction have been observed by others [34]. These differences could for example be driven by heterogenous vascularization or necrosis in distinct tumor areas. Moreover, reduction in diffusion limited but not total hypoxia in B16ova shows that this factor should be taken into account when selecting patients for OXPHOS inhibition.

OXPHOS inhibition resulted in a shift of tumor cell metabolism towards glycolysis. This shift is indicated by an increased ECAR of tumor cells and an increased glucose uptake as measured by [^18^F]FDG uptake in IACS-010759 treated cancer cells and spheroids. This shift in metabolism is necessary to maintain stable intracellular ATP levels under conditions where OXPHOS cannot be utilized. Such an increase of [^18^F]FDG uptake has been described in relation to the OXPHOS inhibitor metformin in mice and patients [35], [36]. Moreover, an increased sensitivity to OXPHOS inhibition under low glucose conditions and a decreased sensitivity under hypoxic conditions indicate such metabolic rewiring, as cells are expected to rely on OXPHOS when glucose is scarce, but use glycolysis when nutrients are plentiful [37]. One of the putative mechanisms by which rewiring is mediated is an increased phosphorylation of the energetic stress sensor AMPK that is observed in this and other studies [38]. The metabolic plasticity displayed by cancer cells suggests the inhibition of OXPHOS as a monotherapy, as suggested for some tumor types relying solely on OXPHOS, might be ineffective for other indications and potentially even promote glycolysis fueled proliferation [30], [39]. Simultaneously targeting OXPHOS and glycolysis might resolve this issue, but this is expected to further increase systemic adverse effects [40], [41]. Additionally, blockade of glutamine metabolism could be considered as an alternative strategy as it simultaneously reduces OXPHOS and glycolysis, subsequentially reducing hypoxia and improving immunotherapy response [42].

OXPHOS inhibitors tested in this study are not selective for tumor tissue, and mice treated with IACS-010759 show elevated serum lactate levels and increased [^18^F]FDG uptake in lung, stomach, spleen and liver. This is in line with earlier reports of lactic acidosis in mice and patients [43], [44]. Interestingly, [^18^F]FDG uptake in the brain of mice was significantly decreased following IACS-010759 treatment, indicating less plasticity and greater dependence on OXPHOS of the brain compared to other organs [45]. This might partly explain the neurotoxicity observed in a recent clinical trial where patients with advanced solid malignancies and acute myeloid leukemia were treated with several IACS-010759 dosing schemes [44]. Severe adverse effects observed in this trial, and effects observed in mice in combination with isoflurane anesthesia, render IACS-010759 unsuitable for clinical use. Future studies should therefore focus on the use of less potent OXPHOS inhibitors that have shown hypoxia reduction in clinical trials, such as metformin or atovaquone [46], [47]. Some studies have already reported an increase in efficacy of radiotherapy or immunotherapy in combination with metformin, although overall results remain inconsistent so far and no data from randomized studies are available [48], [49]. It has to be noted that most of these studies do not stratify patients for diffusion limited hypoxia and are therefore possibly overlooking a beneficial effect in a subpopulation of patients [50]. Additionally, a more targeted delivery of these drugs could be investigated, for instance through mitochondrial targeted variants that have potentially less off target toxicities [22], [51].

In several studies increased OXPHOS has been linked to hypoxia and an immunosuppressive phenotype [[6], [7], [8]]. Reports of increased immune- and/or radiotherapy efficacy following OXPHOS inhibition in mice models exist, presumably through an alleviation of hypoxia, and increased anti-tumor immune response induced by radiotherapy could even further enhance immunotherapy efficacy [8], [18], [19], [52]. In the models used we have confirmed a lower presence of immune cells in hypoxic areas. However, in this first insight we have not observed a change in the presence or spatial distribution of leukocytes following 4 days of OXPHOS inhibition in this study. Further analysis into distribution of specific immune cell subsets and their functionality might reveal different results and should be the focus of future studies. Other explanations for the absence of an effect on immune cells might include timing of our analysis, a direct inhibitory effect of OXPHOS inhibition on immune cells that rely on OXPHOS during several stages of their lifespan, or an inhibitory effect by acidification of the environment through increased glycolysis [53], [54]. Use of less potent inhibitors, different timing of drug administration or different dosing (e.g. prolonged periods at lower doses) may result in an increase of immune cell presence and function as is observed in other studies using metformin [18], [55].

In conclusion, we show that the OXPHOS inhibitor IACS-010759 induces a metabolic shift in tumor cells and other tissues. Furthermore, inhibition of OXPHOS leads to a reduction of hypoxia in 3D cell culture and immunocompetent mouse models, which could potentially lead to increased cancer therapy efficacy in future studies. However, caution is warranted when using potent OXPHOS inhibitors like IACS-010759 as systemic metabolic rewiring can cause adverse effects.

## CRediT authorship contribution statement

**Daan F. Boreel:** Conceptualization, Formal analysis, Investigation, Writing – original draft. **Anne P.M. Beerkens:** Formal analysis, Investigation, Writing – review & editing. **Sandra Heskamp:** Conceptualization, Funding acquisition, Supervision, Writing – review & editing. **Milou Boswinkel:** Investigation, Writing – review & editing. **Johannes P.W. Peters:** Investigation, Writing – review & editing. **Gosse J. Adema:** Conceptualization, Supervision, Writing – review & editing. **Paul N. Span:** Conceptualization, Supervision, Writing – review & editing. **Johan Bussink:** Conceptualization, Funding acquisition, Supervision, Writing – review & editing.

## Declaration of Competing Interest

The authors declare that they have no known competing financial interests or personal relationships that could have appeared to influence the work reported in this paper.
