# Supplementary material for: Inhibition of OXPHOS induces metabolic rewiring and reduces hypoxia in murine tumor models
Source: Clin Transl Radiat Oncol. 2024 Oct 14;49:100875. doi: 10.1016/j.ctro.2024.100875 (PMC11513494; doi:10.1016/j.ctro.2024.100875)
Supplement: Supplementary Data 1 [file mmc1.docx]

**Supplementary figures**


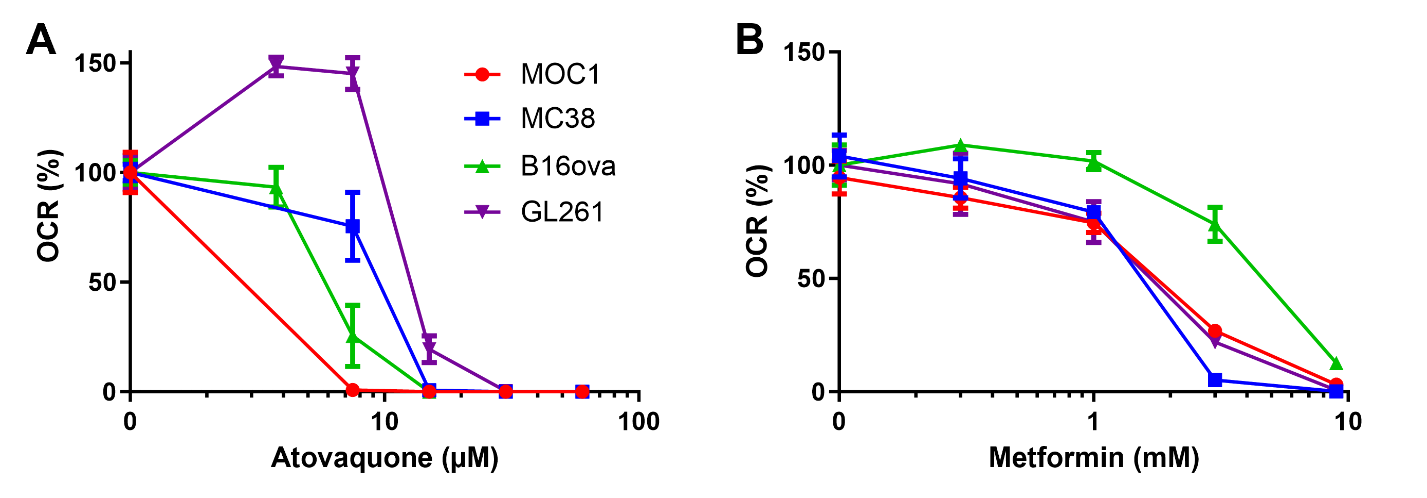


**Supplementary figure S1.** Basal respiration (% OCR) of several murine cancer cell lines treated with atovaquone (A) or metformin (B) for 24 h.


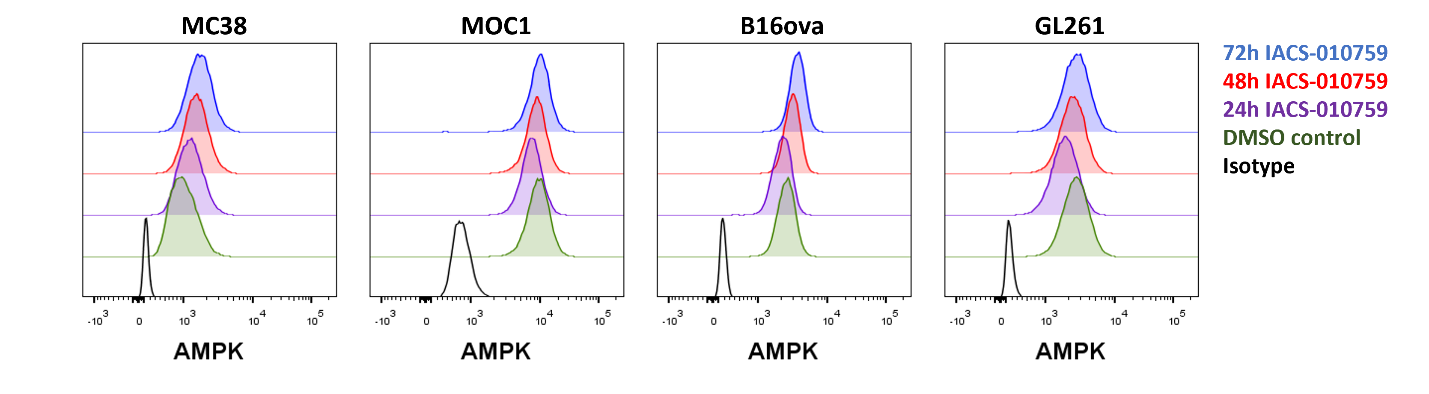


**Supplementary figure S2.** AMPK expression in several murine cancer cell lines treated untreated or treated with IACS-010759 (1.0 μM) for 24, 48 and 72 h.


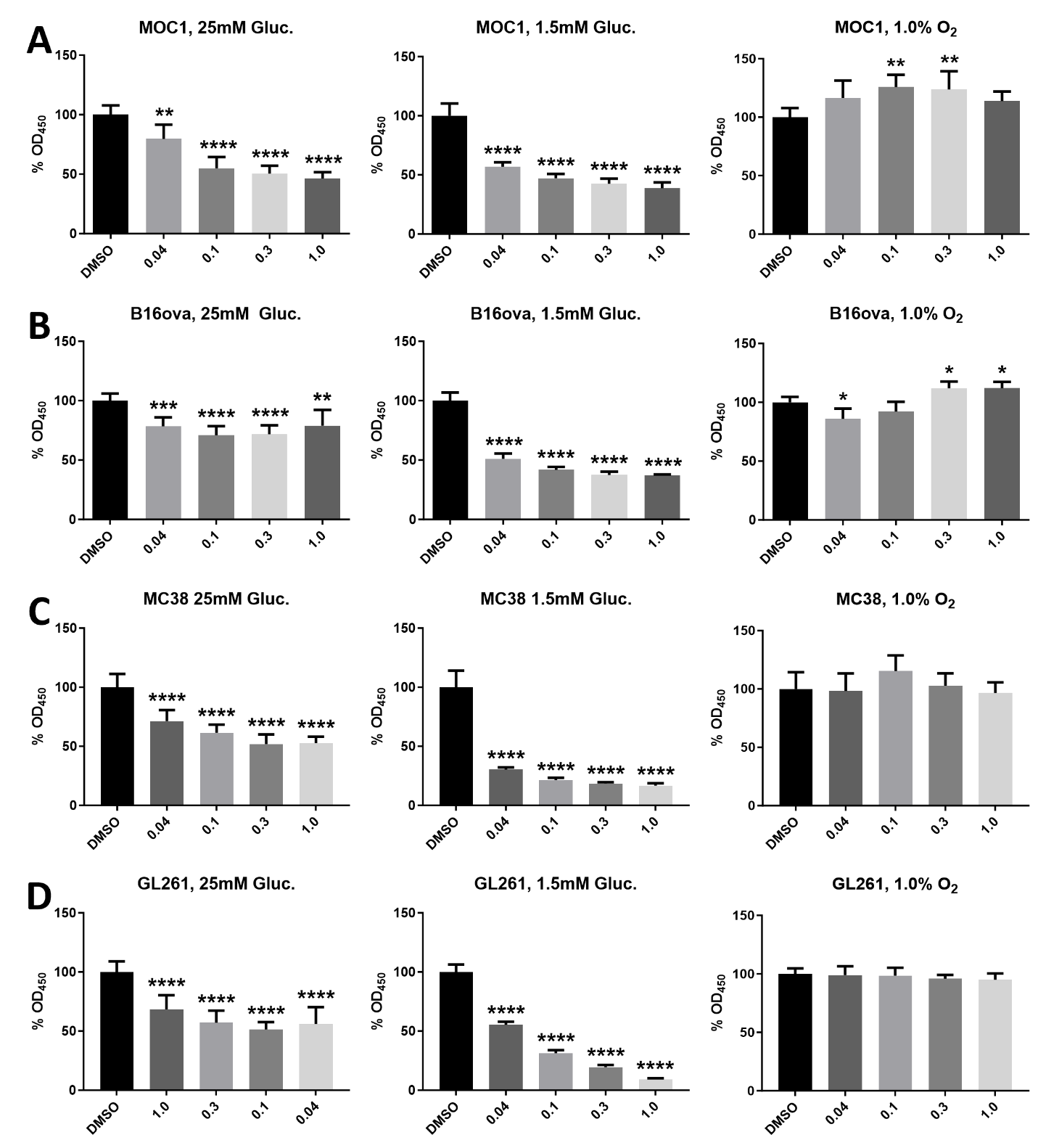


**Supplementary figure S3.** Metabolic activity of (A) MOC1, (B) B16ova, (C) MC38 and (D) GL261 under high glucose (25 mM), low glucose (1.5 mM) and hypoxic (1.0 % O_2_, 25mM Glucose) from samples of figure 2 measured by CCK-8 assay.


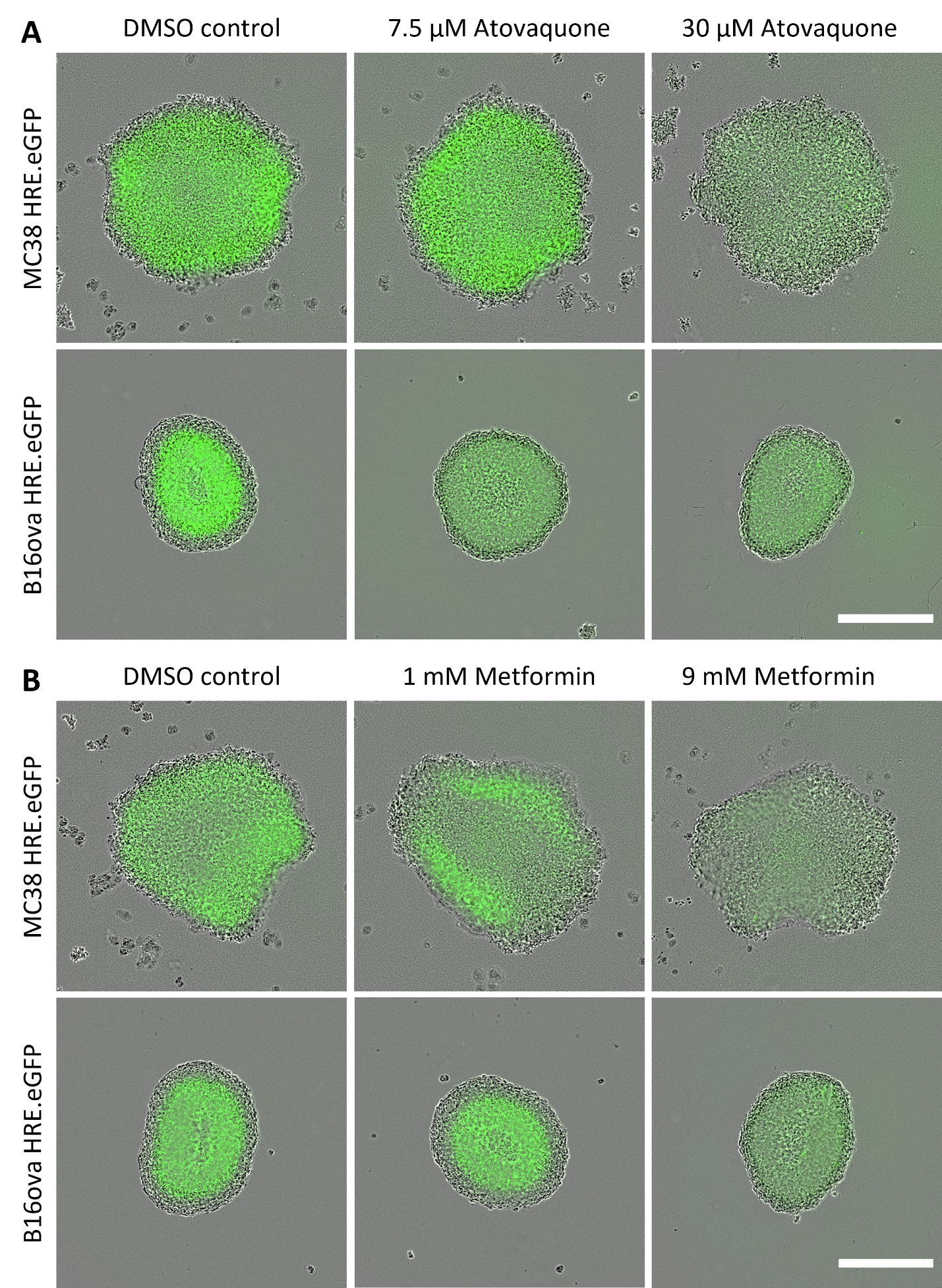


**Supplementary figure S4**. Fluorescent images of MC38 HRE.eGFP and B16ova HRE.eGFP spheroids 24 h post treatment with (A) atovaquone and DMSO or with (B) metformin and DMSO. Scalebar represents 500 μm.


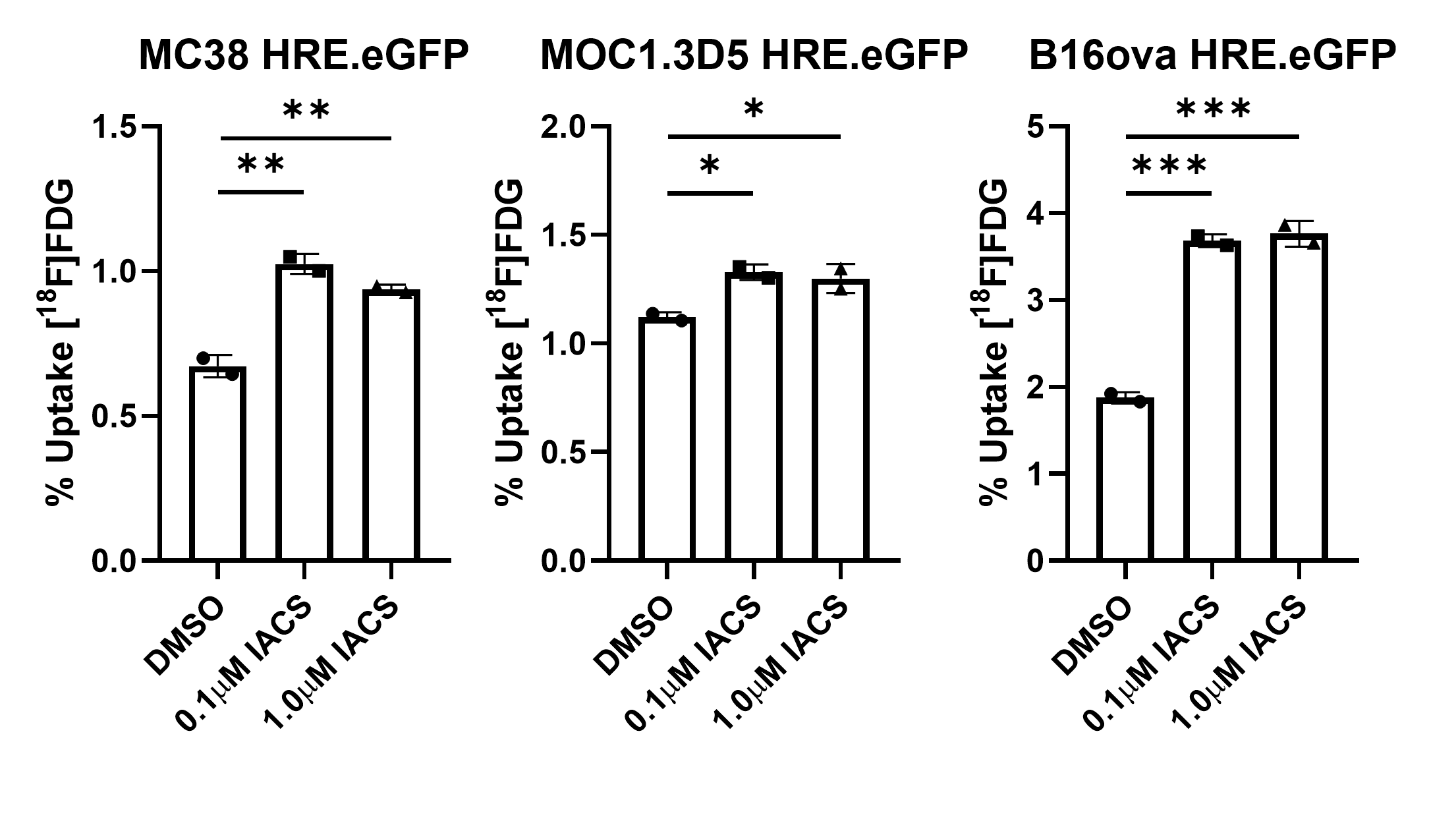
**Supplementary figure S5.** Uptake of [^18^F]FDG in MC38 HRE.eGFP, B16ova HRE.eGFP and MOC1.3D5 HRE.eGFP cells 3 h post IACS-010759 or DMSO treatment.


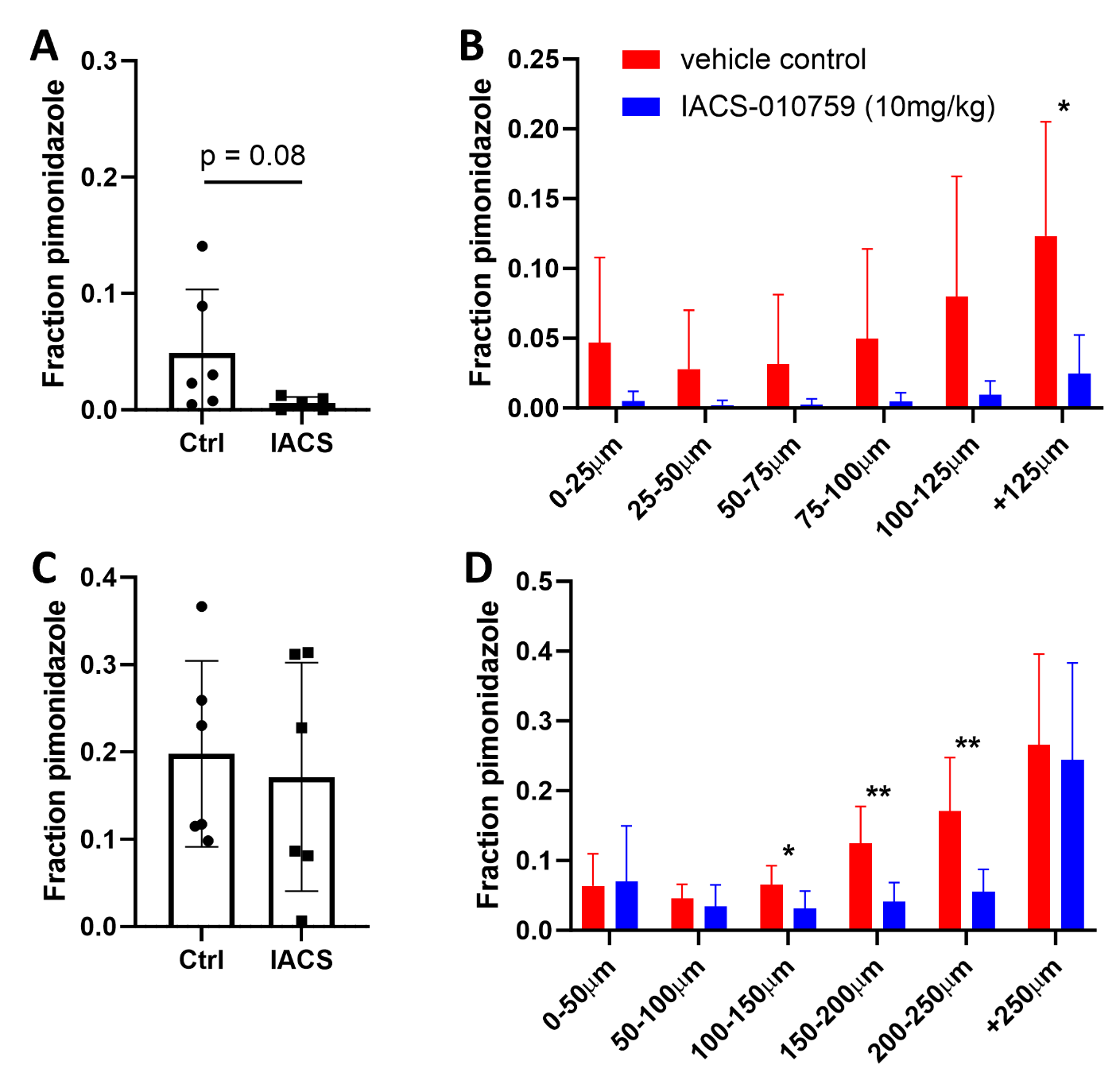


**Supplementary figure S6.** Inhibition of OXPHOS decreases hypoxia in MC38 and B16ova tumors. (A) Fraction pimonidazole positive area on MC38 whole tumor sections of mice treated with IACS-010759 (10 mg/kg) or vehicle control for 4 consecutive days, n = 6. (B) Pimonidazole positive fractions at binned distances to perfused vasculature of A. (C) Fraction pimonidazole positive area on B16ova whole tumor sections of mice treated with IACS-010759 (10 mg/kg) or vehicle control for 4 consecutive days, n = 6. (D) Pimonidazole positive fractions at binned distances to perfused vasculature of C.


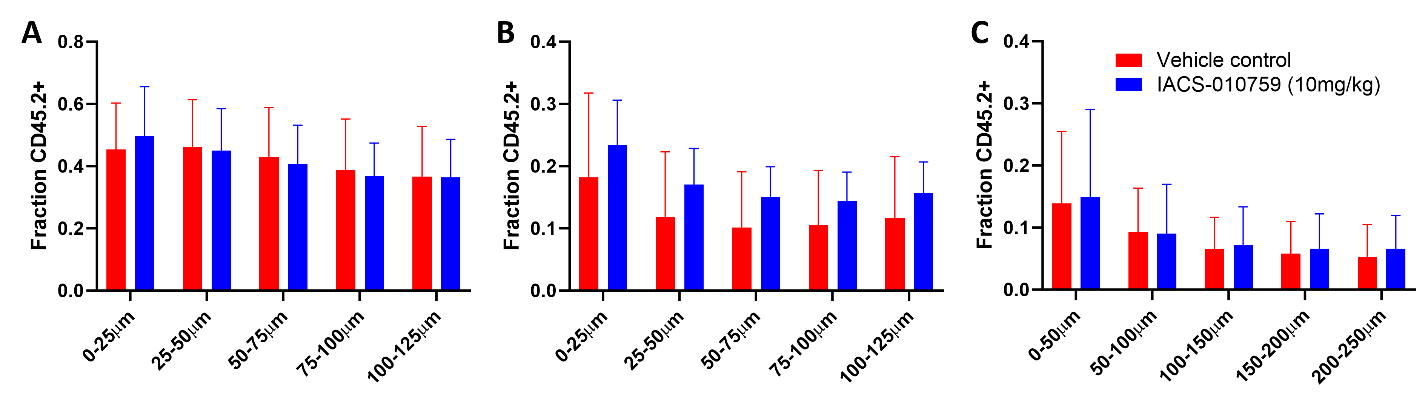
**Supplementary figure S7.** Inhibition of OXPHOS does not change immune cell localization relative to perfused vasculature. CD45.2 positive fractions at binned distances to perfused vasculature on tumor sections of mice bearing (A) MOC1.3D5 (n = 6), (B) MC38 (n = 5) or (C) B16ova (n = 6) tumors treated with IACS-010759 (10 mg/kg) or vehicle control for 4 consecutive days.

|  |  |  |
| --- | --- | --- |
| **Tissue** | **Vehicle control**  **(%ID/g)** | **IACS-010759 (%ID/g)** |
| **Blood** | 0.57 ± 0.14 | 0.52 ± 0.05 |
| **Muscle** | 2.75 ± 1.22 | 3.85 ± 1.73 |
| **Lung** | 3.03 ± 0.26 | 4.31 ± 0.61** |
| **Heart** | 18.30 ± 7.20 | 19.71 ± 3.23 |
| **Brain** | 6.50 ± 0.60 | 5.29 ± 0.90* |
| **Kidney** | 1.09 ± 0.06 | 1.30 ± 0.26 |
| **Liver** | 0.80 ± 0.05 | 0.88 ± 0.06* |
| **Stomach** | 3.33 ± 0.98 | 5.99 ± 0.99** |
| **Colon** | 3.77 ± 0.41 | 3.26 ± 0.45 |
| **Spleen** | 2.32 ± 0.15 | 3.48 ± 0.63** |
| **Small intestine** | 1.74 ± 0.30 | 1.85 ± 0.35 |
| **Tumor draining LN** | 4.32 ± 2.81 | 3.34 ± 1.21 |
| **Non-draining LN** | 2.94 ± 0.43 | 3.72 ± 2.20 |
| **Tumor** | 6.71 ± 1.46 | 6.76 ± 1.11 |
| **Blood glucose (mM)** | 8.66 ± 0.68 | 10.08 ± 1.54 |

**Supplementary Table S1.** *Ex vivo* biodistribution of [^18^F]FDG uptake and blood glucose (before anesthesia) levels in C57BL/6 mice with subcutaneous B16ova (n = 5) tumors. Mice were treated for 4 consecutive days with IACS-010759 (10 mg/kg) or vehicle control. Lymph node; LN.
